# MiR-33a plays an crucial role in the proliferation of bovine preadipocytes

**DOI:** 10.1080/21623945.2021.1908655

**Published:** 2021-04-12

**Authors:** Wenzhen Zhang, Li Wang, Sayed Haidar Abbas Raza, Xiaoyu Wang, Guohu Wang, Chengcheng Liang, Gong Cheng, Bingzhi Li, Linsen Zan

**Affiliations:** a College of Animal Science and Technology, Northwest A&F University, Yangling, Shaanxi, P.R. China; b National Beef Cattle Improvement Center, Northwest A&F University, Yangling, Shaanxi, P.R. China

**Keywords:** miR-33a, bovine preadipocytes, proliferation, CDK6

## Abstract

Preadipocyte proliferation is a critical and precisely orchestrated procedure in adipogenesis, which is highly regulated by microRNAs (miRNAs). A previous study identified that the expression of miR-33a is different in intramuscular fat (IMF) tissues from steers and bulls. In the present study, miR-33a was overexpressed in bovine preadipocytes, and a total of 781 differentialy expressed genes were found, including 348 upregulated and 433 downregulated genes. Gene Ontology and Kyoto Encyclopaedia of Genes and Genomes (KEGG) pathway analyses of the differentially expressed genes enriched cell division and cell cycle respectively. MiR-33a overexpression decreased the rate of preadipocyte proliferation. Synchronously, the mRNA and protein expression levels of proliferation-related marker genes, including cyclin B1 (CCNB1) and proliferating cell nuclear antigen (PCNA), were decreased. In contrast, inhibiting miR-33a increased the rate of preadipocyte proliferation, and expression levels of CCNB1 and PCNA. Furthermore, based on luciferase reporter assays, miR-33a targeted directly cyclin-dependent kinase 6 (CDK6)-3ʹUTR and inhibited CDK6 protein expression. Interestingly, the silencing of CDK6 inhibited bovine preadipocyte proliferation and proliferation-related genes. Therefore, miR-33a inhibits the proliferation of bovine preadipocytes. CDK6 is the target gene of miR-33a and may be involved in the effects of miR-33a on bovine preadipocyte proliferation.

## 1. Introduction

Adipocytes are mainly involved in energy storage. There are two main ways by which adipose tissues increase in size: one is hypertrophy, and an increase in the size of existing adipocytes; the other is hyperplasia, the formation of new adipocytes through the proliferation and differentiation of resident precursors known as preadipocytes [1]. Recent studies have acknowledged the effect of adipocytes on adverse metabolic consequences, such as insulin resistance, dyslipidemia, hepatic steatosis, coagulopathies, and hypertension [2–4]. Additionally, adipocyte precursor proliferation may be involved in the wound-healing process [5]. In beef cattle, the adipose site and content, focused on proliferation and differentiation, affect meat flavour, juiciness, tenderness, and colour.

miRNAs are a class of endogenous small non-coding RNAs composed of approximately 22 nucleotides and play an essential role in post-transcriptional regulation by target mRNA degradation or translation inhibition [6]. miRNAs have hundreds of potential target genes that can participate in complex molecular regulatory networks [7]. MiR-143 was the first miRNA found to be related to adipocytes; it is involved in regulation of adipocyte differentiation by target gene extracellular signal-regulated kinase 5 (ERK5) [8].

Subsequently, several miRNAs have been demonstrated to regulate adipose proliferation and differentiation. For instance, miR-2400 could promote bovine preadipocyte proliferation by directly targeting PR/SET domain 11 (PRDM11) [9]. MiR-145, classified into the same cluster as miR-143, was found to inhibit adipogenesis in bovine preadipocytes by reducing the activity of PI3K/Akt and MAPK signalling pathways [10]. Similar to the previously mentioned miRNAs, miR-93, miR-17-92, miR-27, miR-378, and miR-130 have been reported to regulate proliferation and differentiation of preadipocytes [11–14]. MiR-33a is located in the sterol-regulatory element-binding protein transcription factor 2 (SREBF2) gene of chromosome 5. A considerable number of studies have demonstrated that miR-33a regulates the proliferation of cancer cells in colorectal cancer, gastric cancer, lung cancer, osteosarcoma, and breast cancer. In a previous study, miR-33a expression was significantly higher in IMF tissues from bulls than in steers, while castration could alter adipogenesis and increase the IMF of bovine [15]. Interestingly, many studies have indicated that the expression of adipogenesis-related miRNAs is altered in adipogenesis. Thus, miR-33a may be involved in adipogenesis regulation. CDK6, fitted to cdc2-related kinases and first identified in 1994 [16], was known as a classic cell cycle kinase with the highly homologous enzyme CDK4 and played key roles in cell proliferation [17]. The aim of this study is to identify the molecular mechanisms underlying miR-33a in bovine preadipocytes.

## 2. Materials and methods

### 2.1 Cell culture and transfection

Bovine preadipocytes were isolated using a previously described method [18]. The bovine preadipocyte cell culture was maintained in a medium containing 89% DMEM/F12 (Hyclone, USA), 10% foetal bovine serum (PAN-Biotech, Germany), and 1% penicillin/streptomycin at 37°C with 5% CO_2_. After growth to 50% confluence, the cells were transfected with miR-33a mimic (50 nM), negative control (NC) (50 nM), inhibitor (100 nM) and inhibitor negative control (inhibitor NC) (100 nM), using Lipofectamine 3000 reagent (Thermo Fisher Scientific, USA) according to the manufacturer’s instructions.

### 2.2 Analyses of Differentially Expressed Genes (DEGs) and pathway enrichment

After 48 h of transfection with the miR-33a mimic, NC, bovine preadipocytes were harvested. RNA-Seq was performed by Novogene using the Novaseq platform (Illumina, CA, USA). From the raw data for the six samples (three biological replicates of each group), clean reads were mapped to the bovine genome (Bos_taurus.UMD3.1, release-94, Ensembl database) using Hisat2 [19]. Fragments Per Kilobase of transcript sequence per Millions (FPKM) was an effective tool to quantify gene expression for RNA-seq [20], and FPKM values of annotated genes were measured with featurCounts in the study [21]. DEGs were determined using the DESeq2 R package, with the criterion of a |log_2_ fold change (log_2_FC)| >0.5 and padj < 0.05 [22]. Gene Ontology (GO) term and Kyoto Encyclopaedia of Genes and Genomes (KEGG) pathway analyses were performed using clusterProfiler [23,24], with the criterion of a padj < 0.05.

### 2.3 EdU and flow cytometry assay

To explore the effects of miR-33a on the proliferation of bovine preadipocytes, the 5-ethynyl-2ʹ-deoxyuridine (EdU) assay was performed using a cell light EdU DNA proliferation kit (RiboBio, Guangzhou, China). Bovine preadipocytes were seeded in 24-well cell culture plates and transfected at 50% density with miR-33a mimic NC inhibitor and inhibitor NC respectively. After 48 h, EdU staining was performed on the cells according to the manufacturer’s instructions. Briefly, bovine preadiocytes were seeded in 6-well cell culture plates and transfected as described above. Cells were then stained with 4ʹ, 6-diamidino-2-phenylindole (DAPI) and detected by flow cytometry.

### 2.4 Quantitative real-time PCR (qRT-PCR)

Total RNA was extracted from cells using RNAiso Plus (Takara, Dalian, China) according to the manufacturer’s instructions. Reverse transcription of miRNA and mRNA was performed using the miRcute Plus miRNA First-Strand cDNA kit (TianGen, Beijing, China) and Prime Script TMRT reagent Kit with gDNA Eraser (Takara, Dalian, China). Real-time PCR was performed using miRcute Plus miRNA qPCR Detection Kit (TianGen) and TB Green Premix Ex Taq (Tli RNaseH Plus) (Takara) on the CFX connect system (Bio-Rad, USA), following the manufacturer’s instructions. Relative quantification was achieved by normalizing the quantity of U6 and β-actin genes, using the 2^−∆∆Ct^ method [25].

### 2.5 Western blotting

Western blot analysis was performed as described previously [26]. Western blot was imaged using a chemiluminescent horseradish peroxidase substrate (Millipore, Bedford, USA) on a Gel Doc^TM^ XR+ System with Image Lab^TM^ Software (Bio-Rad, USA). The antibodies used in this study were as follows: CCNB1 antibody (55,004-1-AP, 1:500, Proteintech, Rosemont, USA), CCND2 antibody (10,934-1-AP, 1:500, Proteintech), PCNA antibody (10,205-2-AP, 1:2000, Proteintech), and β-actin antibody (6008-1-Ig, 1:5000, Proteintech), and two secondary antibodies (ab150113 and ab150077, 1:5000, Abcam, Cambridge, UK). Quantification of the western blot was performed using the Image Lab (Bio-Rad).

### 2.6 Dual-luciferase reporter assay

The sequences of CDK6-3ʹUTR and its consistent mutation were designed, synthesized, and inserted into the luciferase reporter vector psi-CHECK2 vectors (Promega, USA) between *Xho* I and *Not* I restriction sites, termed CDK6–3ʹUTR-wt and CDK6-3ʹUTR-mut, respectively. Both plasmids were co-transfected with the miR-33a mimic or NC into HEK-293 T cells. The relative luciferase activity was examined using the Dual-Luciferase Assay Kit (Promega, USA) according to the manufacturer’s instructions.

### 2.7 Statistical analysis

The data were analysed using SPSS (version 18.0 IBM, SPSS, Chicago, USA) and GraphPad Prism 6.01 (San Diego, CA, USA). All quantitative experiments were performed three times, and the Student’s t-test was performed to analyse whether the two groups were statistically significance. The results are presented mean ± SD. For all analyses, group differences were considered statistically significant at P < 0.05 (*, significant) or P < 0.01 (**, extremely significant).

## 3. Results

### 3.1 Identification and enrichment analyses of DEGs associated with miR-33a

**Figure 1.**
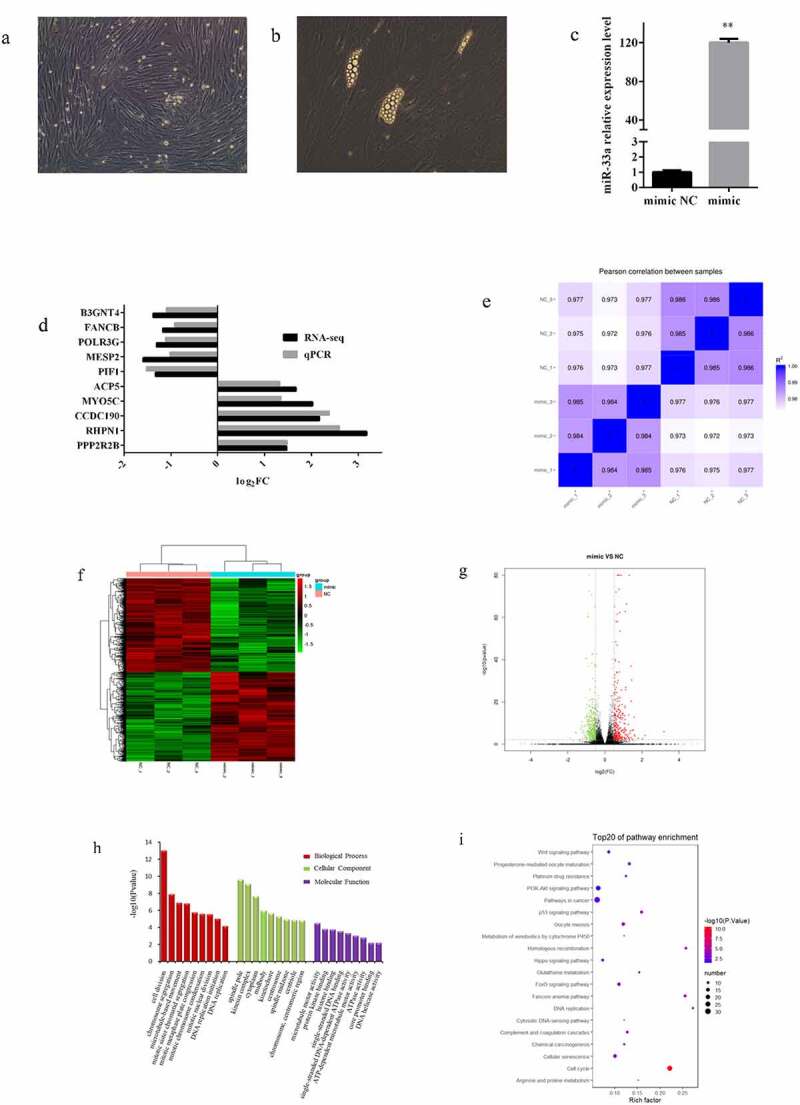
**Identification and enrichment analysis of DEGs associated with miR-33a**. (a) Bovine preadipocytes. (b) Bovine preadipocytes were induced to differentiate for 12 days. (c) The overexpression efficiency of miR-33a was measured by qRT-PCR after 48 h of transfection with miR-33a mimic and NC into bovine preadipocytes (n = 3). (d) Ten randomly selected DEGs were analysed using qRT-PCR, and the results were compared with those of RNA-seq (e) Correlation analysis among sequencing samples according to the gene FPKM date. (f) Heatmap for hierarchical cluster analysis of all sequencing samples and DEGs. Red represents relatively high expression, and green represents low expression. (g) Volcano plot for the distribution of DEGs. Red and green represent upregulated and downregulated genes, respectively. (h) GO enrichment analysis result for DEGs. (i) Top 20 pathways of KEGG enrichment analysis result for DEGs. * *P* < 0.05, ** *P* < 0.01

To identify the bovine preadipocytes isolated, the cells were induced differentiation and formed lipid droplets (Figure 1(a, b)). MiR-33a mimic and NC were transfected into bovine preadipocytes to analyse the potential role of miR-33a in bovine preadipocytes via extensive sequencing analysis. After 48 h of transfection, total RNA was extracted from the cells and reverse transcribed. The expression of miR-33a was upregulated 120-fold in the mimic group (Figure 1c). The cells were subjected to RNA-seq. Ten randomly selected DEGs were verified by qRT-PCR, and the results were consistent with RNA-Seq analysis (Figure 1d). After expression quantification, correlation analyses were performed among samples, and the results were consistent with the grouping of the samples (Figure 1e). A heat map was used to display the clustering of the samples and DEGs (Figure 1f). Differential expression analysis was performed, with 781 DEGs (348 upregulated and 433 downregulated genes) identified in the mimic group (Figure 1g). Pathway analyses of DEGs were conducted to understand the pathways and molecular interactions involved in miR-33a. The significant GO terms and KEGG pathways were based on a padj < 0.05, calculated by the hypergeometric test. The top significant GO term enrichment analysis and the top 20 KEGG pathway enrichment analysis demonstrated a cellular response centred on the axis of proliferation processes (Figure 1(h, i)). The GO term contained cell division and mitotic-related processes, and the KEGG enrichment pathways contained cell cycle, DNA replication, and PI3K-Akt signalling pathways. The results of the enrichment analyses suggested that the miR-33a may be involved in the proliferation of bovine preadipocytes.

### 3.2 Expression of miR-33a in tissues and cells

**Figure 2.**
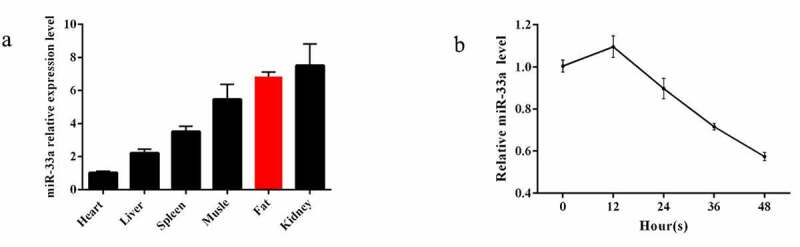
**Tissue and cellular expression of miR-33a** (a)Expression profile of miR-33a in differential six tissues (heart, liver, spleen, muscle, fat, and kidney) (n = 3). (b) Expression of miR-33a during bovine preadipocyte proliferation (0, 12, 24, 36, and 48 h) (n = 3)

Numerous miRNAs have been identified that are altered during adipocyte proliferation, and many of these have been demonstrated to be essential regulators of proliferation. To determine whether miR-33a is associated with bovine preadipocyte proliferation, its expression was measured in six different tissues and different time of preadipocyte proliferation by qRT-PCR in the initial study. The highest miR-33a expression was found in tissues of the kidney, followed by fat, muscles, tissues of spleen, and tissues of liver, and the lowest expression level was found in the heart (Figure 2a). During preadipocyte proliferation, the highest miR-33a expression was found at 12 h, and the lowest was found at 48 h, and the expression of miR-33a decreased substantially (Figure 2b). These findings suggest that miR-33a is a candidate regulator of preadipocyte proliferation.

### 3.3 Overexpression of miR-33a inhibited bovine preadipocyte proliferation

**Figure 3.**
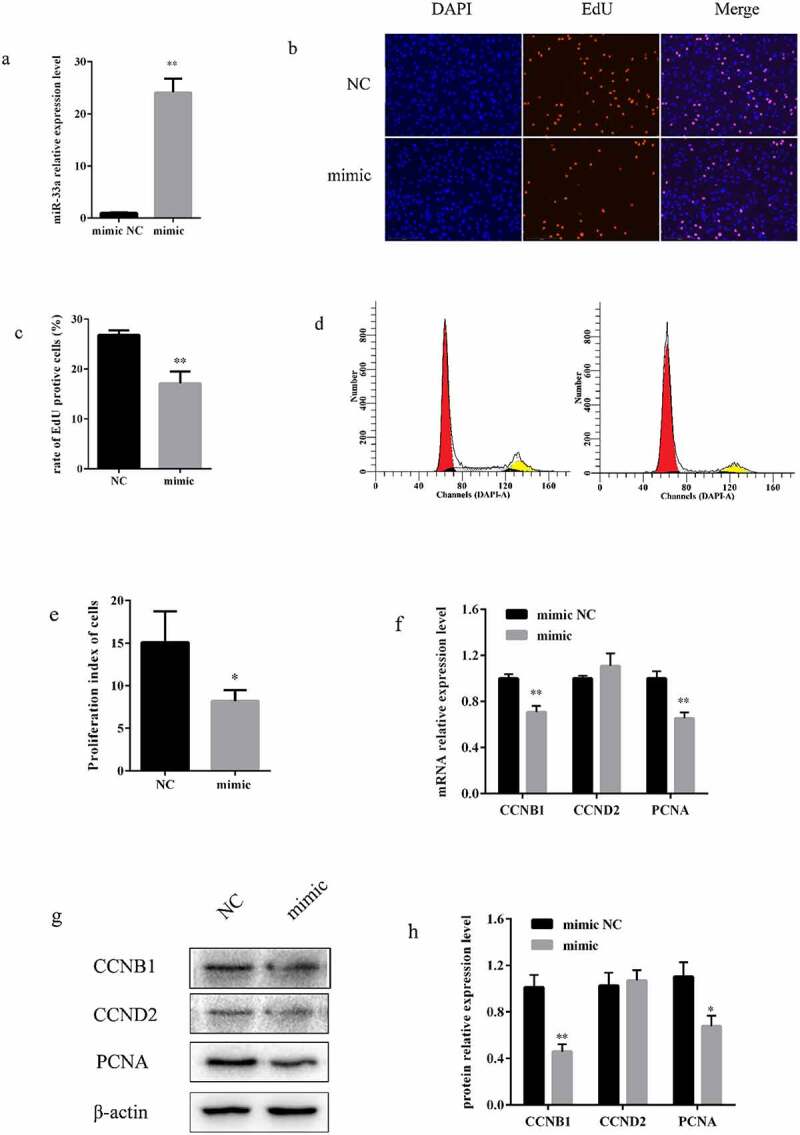
**Overexpression of miR-33a inhibited bovine preadipocyte proliferation**. (a) The overexpression efficiency of miR-33a was measured by qRT-PCR after 48 h of transfection with miR-33a mimic and NC into bovine preadipocytes (n = 3). (b) EdU proliferation assay after 48 h of transfection with miR-33a mimic and NC (n = 3). (c) The percentage of EdU-positive cells (EVOSTM Auto 2, 200×). (d) Flow cytometry assay, performed after 48 h of transfection with mimic (right) and NC (left) (n = 3). (e) Statistical data of the flow cytometry assay. (f) Relative mRNA expression of CCNB1, CCND2, and PCNA genes after 48 h of transfection with miR-33a mimic and NC (n = 3). (g) Protein expression analysis of CCNB1, CCND2, and PCNA by western blotting. β-actin was used as an internal reference. (h) Statistical data of the protein expression of CCNB1, CCND2, and PCNA. * P < 0.05, ** P < 0.01

To detect miR-33a regulated proliferation, miR-33a mimic and NC were transfected into preadipocytes. MiR-33a expression was upregulated 24-fold in the mimic group compared to the NC group (Figure 3a). EdU results demonstrated that the cell proliferation rate significantly decreased by nearly 40% when transfected with the miR-33a mimic (Figure 3(b, c)). The cell cycle was detected by flow cytometry. The results revealed that overexpression of miR-33a decreased the percentage of preadipocytes in the S phase (Figure 3(d, e)). Additionally, mRNA and protein expression of CCNB1, CCND2, and PCNA was investigated, to verify the effect of miR-33a on preadipocytes. The results demonstrated that overexpression of miR-33a significantly decreased the expression of CCNB1 and PCNA after 48 h of transfection (Figure 3(f- h)). Collectively, these results demonstrate that overexpression of miR-33a inhibit the proliferation of bovine preadipocytes.

### 3.4 Inhibiting miR-33a promoted bovine preadipocyte proliferation

**Figure 4.**
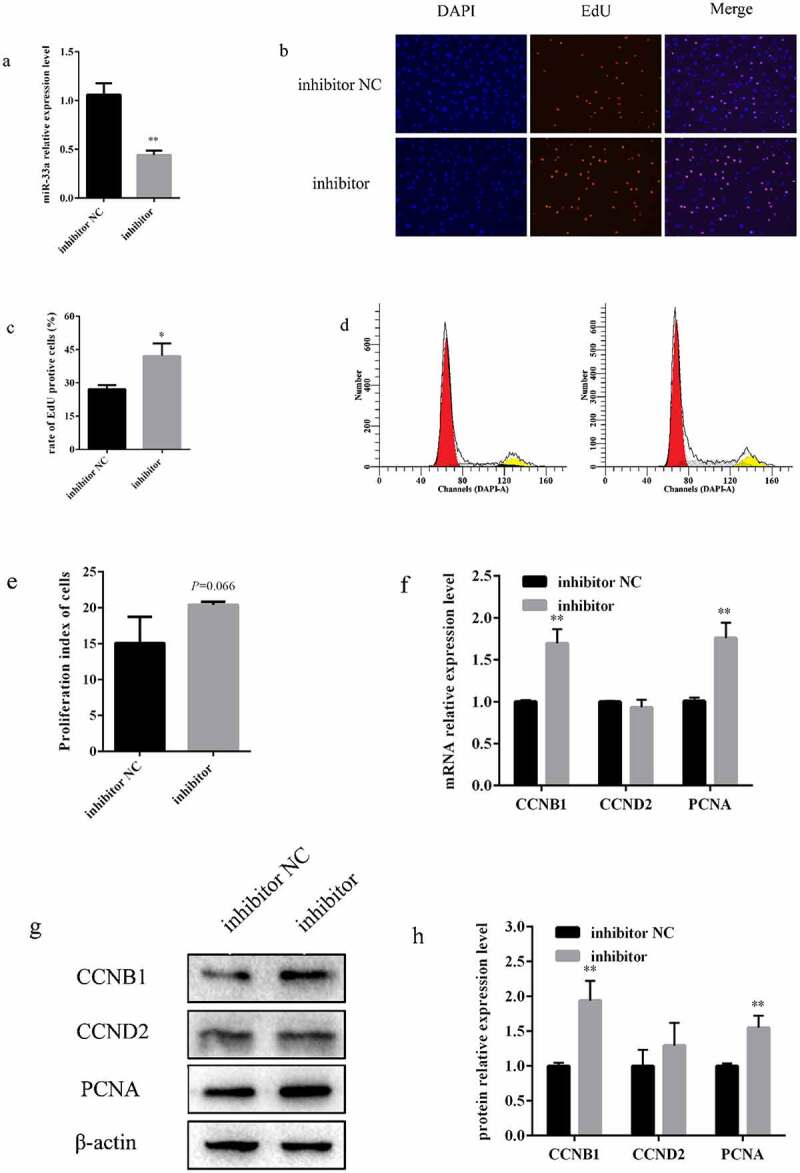
**Inhibiting miR-33a promoted bovine preadipocyte proliferation**. (a) The inhibiting efficiency of miR-33a was measured by qRT-PCR after 48 h of transfection with miR-33a inhibitor and inhibitor NC into bovine preadipocytes (n = 3). (b) EdU proliferation assay, after 48 h of transfection with miR-33a inhibitor and inhibitor NC (n = 3). (c) The percentage of EdU-positive cells (EVOS^TM^ Auto 2, 200 ×). (d) Flow cytometry assay, after 48 h of transfection with inhibitor (right) and inhibitor NC (left) (n = 3). (e) Statistical data of the flow cytometry assay. (f) Relative mRNA expression of CCNB1, CCND2, and PCNA genes after 48 h of transfection with miR-33a inhibitor and inhibitor NC (n = 3). (g) Protein expression analysis of CCNB1, CCND2, and PCNA by western blotting. β-actin was used as an internal reference. (h) Statistical data of the protein expression of CCNB1, CCND2, and PCNA. * *P* < 0.05, ** *P* < 0.01

To explore effect of inhibiting miR-33a on bovine preadipocyte proliferation, miR-33a inhibitor and inhibitor NC were transfected into preadipocytes. miR-33a expression was downregulated by 55% in the inhibitor group compared with that in the inhibitor NC group (Figure 4a). EdU results demonstrated that the cell proliferation rate significantly decreased when cells were transfected with miR-33a inhibitor (Figure 4(b, c)). The cell cycle was detected by flow cytometry. The results displayed that inhibiting miR-33a increased the preadipocyte percentage of in the S phase (Figure 4(d, e)). Additionally, inhibiting miR-33a significantly increased the expression of CCNB1 and PCNA at both the mRNA and protein levels after 48 h of transfection (Figure 4(f-h)). Together, these results demonstrate that inhibiting miR-33a promote the proliferation of bovine preadipocytes.

### 3.5. MiR-33a suppressed CDK6 expression by directly targeting 3ʹ-UTR

**Figure 5.**
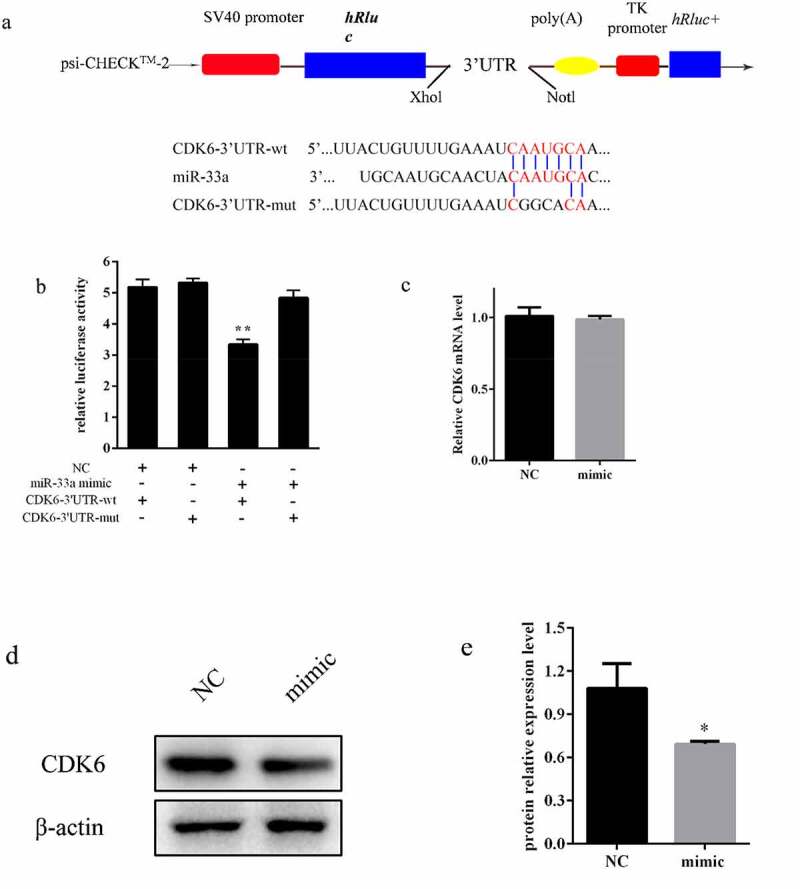
**MiR-33a suppressed CDK6 expression by directly targeting 3ʹ-UTR**. (a) Wild type sequences of miR-33a recognition region predicted by the TargetScan program in the 3′ UTR of CDK6 and its mutant type sequences. The insertion sites of the two sequences in psi-CHECK^TM^-2 with *Xho*I and *Not*I restriction enzyme. (b) Luciferase activities of reporter plasmids with wild type or mutated type CDK6- 3ʹUTR were examined in HEK-293 T cells by cotransfection with miR-33a mimic and NC (n = 3). (c) Relative mRNA expression of CDK6 after 48 h of transfection with miR-33a mimic and NC (n = 3). (d) Relative protein expression of CDK6 after 48 h of transfection with miR-33a mimic and NC. β-actin was used as an internal reference (n = 3). (e) Statistical data of the protein expression of CDK6. * *P* < 0.05, ** *P* < 0.01

CDK6 is a component of the core cell cycle machinery that drives cell proliferation [17]. KEGG analysis suggested that DEGs were significantly enriched in the cell cycle, and CDK6 was predicted to be the target gene of miR-33a using TagetScan (http://www.targetscan.org/vert_71/). To determine whether CDK6 was directly targeted by miR-33a, the wild and mutant type seed sequences of CDK6 3ʹUTR were integrated into psi-CHECK^TM^-2 (Figure 5a) and then transfected into HEK-293 T cells with the mimic or NC together. The miR-33a mimic significantly suppressed luciferase activity of psi-CHECK-2 with CDK6-3ʹUTR-wt compared to the other groups (Figure 5b). Additionally, mRNA and protein expression levels of CDK6 were quantified by qRT-PCR and western blotting, which demonstrated that miR-33a suppressed CDK6 protein expression (Figure 5(d, e)). Therefore, miR-33a directly targeted CDK6 and impacted CDK6 expression at protein level.

### 3.6 Interfering with CDK6 expression inhibited proliferation of bovine preadipocytes

**Figure 6.**
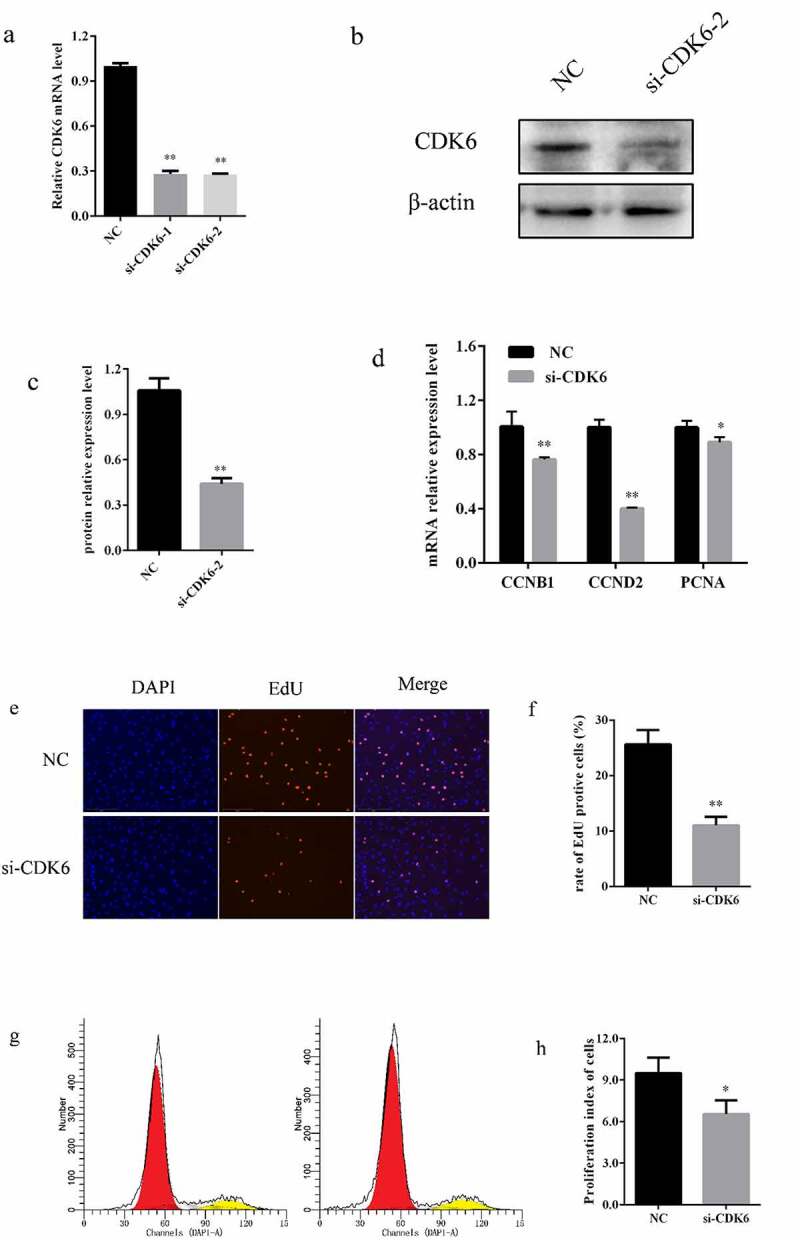
**CDK6 inhibited the proliferation of bovine preadipocytes**. (a) Interference efficiency of si-CDK6-1 and si-CDK6-2, analysed via qRT-PCR after 48 h of transfection. (b) Relative protein expression of CDK6 after 48 h of transfection with si-CDK6-2 and NC. β-actin was used as an internal reference (n = 3). (c) Statistical data of the protein expression of CDK6. (d) Relative mRNA expression of CCNB1, CCND2, and PCNA genes after 48 h of transfection with si-CDK6 and NC (n = 3). (e) EdU proliferation assay after 48 h of transfection with si-CDK6 and NC (n = 3). (f) The percentage of EdU-positive cells (EVOS^TM^ Auto 2, 200 ×). (g) Flow cytometry assay after 48 h of transfection with si-CDK6 (right) and NC (left) (n = 3). (h) Statistical data of the flow cytometry assay. * *P* < 0.05, ** *P* < 0.01

To explore the effect of CDK6 on bovine preadipocyte proliferation, two small interfering RNAs (siRNAs) of CDK6 were synthesized and transfected into preadipocytes. The result demonstrated that both siRNAs had great interference efficiency, reducing approximately 75% of CDK6 mRNA expression and 45% of CDK6 protein expression (Figure 5(a, b, c)), and significantly reduced mRNA expression of functional genes related to proliferation, including CCNB1, CCND2, and PCNA (Figure 6d). Furthermore, interference of CDK6 expression significantly decreased cell proliferation rates by EdU (Figure 6(e, f)) and significantly decreased the percentage of preadipocytes in the S phase (Figure 6(g, h)). Collectively, these results indicate that interfering with CDK6 expression inhibites bovine preadipocyte proliferation.

## 4. Discussion

A low IMF is a challenge to animal science expert, with respect to the improvement of meat quality. Meanwhile, obesity is a global problem that causes diseases, with high associated mortality, in humans. The host gene of miR-33a is SREBF2, which mainly regulates cholesterol-regulatory genes and low-density receptors (LDLRs). MiR-33a is known for controlling cholesterol and lipid metabolism in cooperation with its host gene and has been demonstrated the affect on atherosclerosis in animals by several groups. Among bovines, the expression of miR-33a was higher in intramuscular fat tissues of bulls than steers by transcriptome analyses of miRNAs of Chinese Qinchuan cattle [15]. Moreover, the expression was higher in adipocytes than in preadipocytes of Simmental calves, as determined by small RNA-seq and qRT-PCR analyses [27]. These studies suggest that miR-33a may be involved in the regulation of bovine adipogenesis.

In our study, overexpression of miR-33a identified 781 DEGs, which were enriched in cell proliferation-related pathways, such as cell cycle, p53 signalling pathway, PI3K-Akt signalling pathway, and Wnt signalling pathway. The cell cycle was the most significant among these pathways, according to the P-value. Previous studies have confirmed that cell cycle regulators effectively induce cell proliferation in vitro and in vivo [28]. According to the GO term analyses, DEGs were enriched in several kinds of mitosis-related biological processes, and cell division was the most significant among these processes. A combination of KEGG and GO term analyses of DEGs suggested that miR-33a is very likely involved in the proliferation of bovine preadipocytes. Many studies have contributed to identifying numerous miRNAs, whose expressions are altered in different stages, are essential regulators of adipogenesis [29]. In our study, miR-33a was differentially expressed during the preadipocyte proliferation process, and the trend of relative expression in bovine preadipocytes was consistent with that in duck myoblasts [30]. A combination of EdU, flow cytometry, and western blotting revealed that miR-33a inhibited preadipocyte proliferation. Many studies have demonstrated that miR-33a regulates cell proliferation, especially in cancer cells. Interestingly, miR-33a negatively regulates the proliferation of lung, renal, gastric, melanoma, and osteosarcoma cancer cell [31–35]; but is involved in positive regulation of cancer cells in hepatocellular carcinoma [36]. The aforementioned results are consistent with concert with the KEGG pathway enrichment analysis, where the number of DEGs of pathways in cancer was the largest among the top 20 pathways (Figure 1f). Moreover, miR-33a negatively regulates myoblasts proliferation [30]; but positively regulates the vascular smooth muscle cells [37]. To summarize, miR-33a can regulate the proliferation of different cells with two different effects, even in homotypic cells.

Currently, the dual-luciferase reporter assay is one of the most reliable assays to test the silencing of a predicted target gene by a specific miRNA [38]. In this study, CDK6 was identified as a direct target gene by a dual-luciferase reporter assay, and miR-33a downregulated CDK6 at the protein level but not mRNA level. Early studies of animal miRNAs indicated that translational repression was not accompanied by mRNA destabilization due to the imperfect complementarity of miRNA-mRNA [39]. However, miR-33a could regulates CDK6 at both the mRNA and protein levels in gastric cancer cell [33]. Thus far, it has been difficult to understand miRNA mechanisms in-depth, and the method that made the phenomenon whole and unified has not yet been identified.

It is well known that CDK6 and closely related CDK4 play vital roles in cell proliferation of in mammals, where they contribute to the progression of cells into the S phase of the cell cycle. In the present study, we speculate that interfering with CDK6 suppressed bovine preadipocyte proliferation by a combination of EdU and flow cytometry analyses. In Huh7 and A549 cells, miR-33 regulates cell proliferation by targeting CDK6 and CCND1 [40]. additionally, miR-33 could enhance the premature senescence phenotype and diminish proliferation capacity by targeting CDK6 in mouse embryonic fibroblasts [41]. Therefore, miR-33a inhibited the proliferation of bovine preadipocytes, where CDK6 may be involved in the process as the target gene of miR-33a.

## 5. Conclusion

We conclude that overexpression of miR-33a downregulate 433 genes, and upregulate 348 genes in bovine preadipocytes. The DEGs enriched several biological processes and signalling pathways related to proliferation. MiR-33a inhibits bovine preadipocyte proliferation. CDK6 is the target gene of miR-33a and may be involved in the effects of miR-33a on bovine preadipocyte proliferation.
